# Autopsy-based evaluation of skeletonised human remains: A long-term Indian study

**DOI:** 10.6026/973206300222365

**Published:** 2026-04-30

**Authors:** Brinda Patel, Nidhi Sachdeva Agarwal, Radhika Hande

**Affiliations:** 1 Department of Forensic Medicine and Toxicology, Netaji Subhash Chandra Bose Medical College, Jabalpur, Madhya Pradesh, India; 2 Department of Forensic Medicine and Toxicology, Government Medical College, Seoni, Madhya Pradesh, India

**Keywords:** Forensic autopsy, homicide concealment, human remains, post-mortem interval (PMI), skeletonization

## Abstract

Skeletonized remains pose unique forensic identification challenges, particularly in resource-limited settings where concealment attempts complicate medicolegal investigation. This retrospective descriptive study analyzed 19 skeletonized autopsy cases, assessing gender/age via anthropological methods, PMI from decomposition stage, cause/manner of death from family/police/post-mortem records. Four cases (21%) revealed deliberate concealment through cremation/burial to evade detection, highlighting disposal challenges in Indian contexts. Conventional anthropological techniques proved feasible despite resource constraints, through PMI estimation remained imprecise without advanced tools. Thus, we document patterns of skeletonized cases in India, highlighting infrastructure gaps, documentation needs and the need for multidisciplinary collaboration to achieve optimal medicolegal outcomes.

## Background:

Skeletonization represents the final decomposition phase in cases of complete soft-tissue loss, leaving only bones, which can be either partial or complete [1]. These remains, known as skeletonized remains, are rarely seen in routine cases; they are usually found in rural, forested and remote areas by chance [2]. In many of these cases, vital information about forensic analysis can be lost due to advanced decomposition, environmental exposure and untrained or unprofessional handling [3]. Hence, the comprehensive assessment of these remains has a vital role in the investigation of forensic cases, particularly in cases where bodies are found after a long time of death. Assessment of skeletal remains can help determine the manner and cause of death, the time since death and identity [4]. The existing literature on the Indian context is scarce in explaining the practical aspects, challenges and findings of these examinations in a systematic manner, making it vital to share and record the experience necessary to deal with skeletal remains [5]. This research is the first of its kind to provide a detailed assessment of skeletal remains in the Indian context, examining various skeletal remains at the Indian forensic centre. Previous studies on this issue are case-based [5]. Therefore, it is of interest to analyse and examine the human skeletonized remains for medicolegal autopsy purposes, highlight the practical challenges encountered while assessing these remains and share insights that can help improve reporting, interpretation and handling of skeletal cases in resource-limited settings.

## Materials and Methods:

The present autopsy-based retrospective descriptive study was conducted at the Department of Forensic Medicine and Toxicology, Netaji Subhash Chandra Bose Medical College, Jabalpur, Madhya Pradesh, from 2021 September to 2024 August, spanning three years. The study was conducted after obtaining the necessary approvals from the concerned Institutional committees and in accordance with the ethical guidelines (Letter number IEC/2024/10188). As the research involved analyzing the Institutional records from the medico-legal autopsy of the deceased, informed consent was not required; however, the confidentiality of the subjects was strictly maintained to protect sensitive data.

## Study outcomes:

The primary outcomes were:

[1] Identifying the gender and age of the deceased subject.

[2] Examining the interval of post-mortem

[3] Determining the manner and cause of the death in cases (wherever possible)

[4] Recording the level and condition of skeletonization

[5] Challenges encountered during forensic examination

## Measurement of the outcome:

Outcomes were measured through detailed post-mortem examination, supported by circumstantial evidence, police reports and available information from relatives. Standard anthropological techniques were used to estimate age and sex. The state of remains was recorded to estimate the post-mortem interval. Cause and manner of death were concluded after correlating injury patterns, skeletal trauma, scene findings and lab results where available.

## Study participants:

All skeletonised remains brought to the mortuary for medico-legal autopsy during the study period. Human remains in a complete or partial skeletonised state brought for medico-legal autopsy during the study period were included. Cases where remains were confirmed to be non-human and cases with inadequate documentation or that made examination impossible, were excluded from the study. As this was a retrospective medico-legal study on deceased individuals, informed consent was not applicable. Necessary ethical and legal permissions were obtained from institutional authorities and confidentiality was maintained. Data was recorded from Post-mortem reports, Police case records and inquest papers; crime scene photographs (if provided); and inputs from relatives (where available).

## Procedure:

A total of 19 skeletonised remains were received for medico-legal autopsy over the three-year study period. All cases were referred from peripheral centres lacking qualified forensic experts. Most referrals originated from Mandla district, followed by Balaghat, Katni and Seoni in Madhya Pradesh, India. There was no observed seasonal pattern in the recovery of skeletal remains. All 19 cases of skeletal remains received during the study period were carefully examined using a structured, systematic approach. After receiving the remains at the mortuary, each set was assessed physically and virtually to confirm their human origin by identifying the main anatomic landmarks, including the structure and shape of the vertebrae, long bones, pelvis and skull. Any doubt in the specimens led to their exclusion. After confirmation of human origin, bone cleaning was performed where necessary and the bones were arranged in the correct anatomical order on a flat surface to identify any missing parts or duplications and assess the completeness of the skeleton. The condition of the skeletonized remains was noted in detail, including mummification, the presence of adipocere, foul odour, the level of decomposition, the presence or absence of soft tissues and their state as wet or dry. Any animal gnawing marks, cut marks, fractures and visible trauma were also recorded. To estimate the age of the case at death, multiple skeletal indicators were used, including degeneration changes, fusion of epiphyses in long bones, cranial suture closure and assessment of dental wear and eruption [6]. To assess gender from the remains' skull features, such as the mandible, mastoid process and supraorbital ridges, as well as pelvis features, such as the greater sciatic notch and subpubic angle [7]. Additional bones, such as the humerus and femur, were also evaluated for secondary sexual features, wherever necessary [8]. All the remains were also assessed for other features that could aid identification, including healed fracture sites, anatomical deformities, prosthetic devices and surgical implants in bones. Careful photography and recording were conducted of any belongings found on the remains, such as footwear, jewellery, clothing and other items and their details were cross-checked against information provided by family and police records. PMI (post-mortem interval) was assessed based on the degree of skeletonization, environmental exposure, as suggested by police documents and reports and the condition of the remains [9]. It was divided into three categories: early, immediate and late, corresponding to <1 month, 1-6 months and >6 months, respectively. Final recording for the manner and cause of death was made after correlation of crime scene details to all available data, laboratory results (wherever applicable) and police investigation records. The data collected from all study subjects were recorded in a pre-formatted structured proforma specifically designed for the study. They were later assessed to identify peculiar features, challenges and common patterns. The data were assessed using descriptive statistics and reported as percentages and counts for categorical variables. Considering the size and nature of the data, inferential statistics were not used in the study.

## Results:

Among the 19 cases of skeletonized remains considered for the study, 79% (n=15) were classified as incomplete and a complete skeleton could be identified in 21% (n=4) of the cases assessed. Identification of the remains was possible in 18 of the 19 cases assessed. The deceased subject was identified from police records and from personal belongings, such as footwear, jewellery or clothing, in 15 cases. DNA analysis was required for identity confirmation in 3 cases and 1 case remains completely unidentified. Also, in 1 of the cases, the remains were initially considered female based on gross findings and police records. In contrast, DNA analysis confirmed the gender as male, highlighting the limitations of relying solely on visual assessment. The study results showed that, of 19 cases, the majority of skeletal remains were discovered in the national park or forest area, with 36.84% (n=7) remains, followed by an agricultural field, where 10.53% (n=2) remains were found. Dry well, riverside, nala/open sewer, railway track and cremation ground all contributed to 5.26% (n=1) skeletal remains each. Exhumed remains were found in 10.53% (n=2) cases. In 15.79% (n=3) cases, the place of discovery remained unidentified owing to incomplete/missing documentation and the absence of a history (Table 1 - see PDF). It was seen that for distribution of the skeletonized remains considering skeletonization level, only 26.32% (n=5) cases showed complete skeletonization, complete skeletonization with foul smell was noted in 15.79% (n=3) cases, partial skeletonization was seen in 42.11% (n=8) cases depicting the majority, partial skeletonization with mummification changes and adipocere was seen in 10.53% (n=2) cases and charred remains with fragmented and burnt remains were noted in 0.26% (n=1) case among the 19 cases considered for the present study (Table 2 - see PDF). These results can be attributed to environmental exposure and variation in post-mortem intervals, linked to skeletonized remains and to a focus on the vital role of comprehensive assessment of the stage of decomposition for forensic purposes. In the present study, the post-mortem interval ranged from 1 week to 1 year. In most cases, it was possible to estimate and identify the remains based on environmental exposure and the stage of decomposition. In one case where remains were charred, fragmented and burned, the post-mortem interval was not determined accurately. In 4 cases, the exact cause of death was determined. Two deaths resulted from blunt force trauma to the head, while two others were due to multiple antemortem injuries. In the remaining 15 cases, the cause of death could not be reliably ascertained due to advanced decomposition or incomplete skeletal recovery. The manner of death was determined as homicidal in 2 cases. One involved remains found in a dry well with both hands and feet tied with a ligature and weighted with stones. Another case, involving charred bones, was suspected to be a homicide. This too was confirmed as a homicide case, as revealed by later detailed police investigations. Three cases were suspected suicides two by hanging, where ligature material was retrieved from the scene and one where the body had been buried by family members, reportedly to avoid legal complications. Exhumation of the remains of the dead body was brought in two cases the first involved re-autopsy of five bones after an initial examination at a peripheral centre. The second concerned a road traffic accident in which the body was initially buried without police notification. Police later recovered the body after a delay of a few days. In one case, charred remains were submitted by police after cremation. As per neighbour reports, the deceased, an older man, had allegedly been struck on the head with a metal bucket by his son. The cremation was then done by the son to prove it as a natural death to conceal the crime. Attempts were made to destroy evidence and body disposal in 4 remains, where one was cremated and three were buried. In one case, the body was buried in a shallow grave, which was later exposed to animals. Another case was buried to avoid police involvement after a road traffic accident. The third case was cremated to conceal the homicidal injury and the fourth case was electrocuted and the remains were hidden at a distant place and were recovered after 3 years due to a dispute. These results depict the challenges in forensic analysis, such as concealed, mishandled and incomplete examinations of remains and the limitations of forensic analysis due to advanced decomposition.

## Discussion:

It is difficult to assess the skeletonized remains owing to environmental exposure and soft-tissue loss, making it vital to have sharp practical skills and comprehensive clinical knowledge [10]. The study results showed partial skeletonization in the majority of subjects, with 42% of cases reporting it as common. The second most common pattern was complete skeletonization, occurring in 26% of cases and foul smell accompanied by complete skeletonization in 16% of cases. In 10,53% (n=2) cases, partial skeletonization with signs of mummification and adipocere were seen, depicting immediate decomposition. In 5.26% (n=1) case, charred remains were found. Variation in these findings corresponds to variation in environmental exposure and post-mortem intervals. Mummification and the presence of adipocere were reported late in decomposition, which could be due to limited exposure and localized burial. In contrast, the foul smell could be attributed to recent decay. Cause of death could be reliably established in only 4 of 19 cases (21.05%) in the current study. In the remaining cases, decomposition and soft-tissue loss prevented conclusive findings. Sukumar and Das (2015) [11] also reported similar difficulties in their case, where perimortem trauma was suspected, but lab results were inconclusive. Even with comminuted fractures and weapon correlation, the cause of death remained legally unconfirmed due to the skeletonised state. Braynova *et al.* (2024) described a case with gunshot wounds visible in the skull, but were unable to determine the manner of death due to advanced decay and lack of scene evidence [4]. In the present study, we also noted that such conditions preserved soft tissue and slow decay in some areas while allowing skeletonization in others. Similar variability in decomposition has been reported by Tarantino *et al.* in (2023) [12], who described a case of a partially buried body exhibiting marked differential decomposition. This variability can be due to high environmental variability, partial exposure and/or shallow burial. This was in agreement with Krishna *et al.* [13] from (2013) differential decomposition was recorded in 3 cases, one of which depicted careless dustbin disposal, with bones odourless and dry, indicating prolonged environmental exposure. In another case, soft tissue retention was observed with early decomposition, confirming the variability in decay even within the same residential area. Given the presence of mummification and adipocere, the results of this research were consistent with those of Schotsmans *et al.* [14] in (2011), who reported that adipocere formation occurs under moist, anaerobic conditions. In contrast, desiccation is promoted by low humidity and exposure to air. Both processes coexist in the human body based on environmental insulation and depth of burial. In another case report by Braynova *et al.* [4] in (2024), a complete skeleton with hair and soft tissue remnants was recovered. The post-mortem interval was reported to be 1-2 years; the body had some dried tissue and a greasy texture in the bone. These results favored delayed full skeletonization, based on factors such as microclimate, burial conditions and concealment [15]. he study results showed incomplete remains in 79% of cases (n=15), highlighting that remains are commonly disturbed and damaged before recovery and forensic assessment, which can be attributed to careless handling, environmental exposure and animal activity. This was correlated with a study by Krishna *et al.* [13] in (2013), in which remains were found scattered in the area, usually mixed with animal products or incomplete. In one case, bone separation and animal gnawing resulted in loss of vital anatomical parts, making assessment difficult. One 2024 study by Ohlwärther *et al.* [15] also reported a common finding of fragmented bones compared to a complete skeleton, with incomplete bone observed in 1669 cases from 2781 subjects assessed. Their study identified mechanical recovery injuries and post-mortem animal damage as major factors in the fragmentation of the bone. Identification was possible in 95% cases of the present study. Most of the identification was based on personal items and DNA analysis was conducted on 3 remains. Braynova *et al.* [4] in (2024) reported one case of a fully skeletonised cadaver, in which identity, age and gender were assessed from dentition and anthropological markers.

Similar results were also reported by Ohlwärther *et al.* [15] who observed successful STR profiles in DNA analysis in 81% of their study cases, resulting in the complete identification of 35% of subjects from human remains. These results supported the vital role of combining molecular techniques with anthropological examination, particularly in cases where artefactual or visual identification was uncertain. In this study, forested areas were the most common sites of remains discovery, accounting for 37% of cases, suggesting that the most common disposal sites were secluded environmental areas, either due to isolation, delayed discovery from accidental death, or delayed recovery. Regarding the Indian scenario, a 2013 study by Krishna *et al.* [13] reported similar results, with the recovery of skeletal remains from isolated areas, farmlands and roadsides, with no information about the scene. Another study by Ohlwärther *et al.* [15] suggested that 38% of bones from the study were found in parks, meadows and forests and these are common areas for such findings. Cause of death could be reliably established in only 4 of 19 cases (21.05%) in the current study. In the remaining cases, decomposition and soft- tissue loss prevented conclusive findings. Another study also reported similar difficulties in their case, where perimortem trauma was suspected, but lab results were inconclusive [5]. Even with comminuted fractures and weapon correlation, the cause of death remained legally unconfirmed due to the skeletonised state. Braynova *et al.* (2024) described a case with gunshot wounds visible in the skull, but were unable to determine the manner of death due to advanced decay and lack of scene evidence [4]. In this forensic assessment study, the cause of death manner was concluded for 26.3% (n=5) of the remains, where three were suicidal and two were cases of homicide. The efforts to hide the body were reported in four cases by either cremation or burial. These findings were in line with previous research by Krishna *et al.* [13] who suggested that skeletal remains from criminal involvement are usually discovered in concealed sites and public areas. One case was burned to destroy the body and prevent identification. Another was concealed following a road traffic accident, depicting that skeletal remains are usually a knowingly done attempt to hide the evidence and this adds further complications in forensic examination. In two cases from the present study, exhumation was done, where one remains was reassessed following an incomplete autopsy procedure. For the second case, the discovery of a buried body was done following a road traffic accident, which correlated with Krishna *et al.* who suggested a similar case where initially bones were considered for teaching the students and were confirmed as improper body disposal [13]. These results suggested common concerns, including coordination gaps in forensics and police during early assessment, hasty disposal and poor recordings. The results from the present study depict the challenges and complexities of handling skeletal remains in the forensic context. These results aligned with previous studies conducted in international and Indian settings and highlighted the need for professional training, systematic recovery and the use of multidisciplinary tools, such as comprehensive recordings, anthropology and DNA analysis, to gain insights into forensic matters from skeletonized or degraded remains. The study had a few limitations despite being a strong assessment from the Indian context, including its retrospective nature, which made it solely reliant on post-mortem findings and the records made, thereby compromising data quality, as it was governed by the completeness of the records found during the autopsy. In a few cases, details from the scene and police data were either unclear or missing, which might have compromised the accuracy of the interpretation. The study assessed only 19 remains, resulting in a small sample size over 3 years, which limited statistical analysis and the ability to draw general conclusions. The study considered only cases from medicolegal autopsies and excluded remains handled differently, limiting the general applicability of the results. To reduce observer bias, all remains were assessed using the standard method and cross-verification of results was conducted against police records and details available at the scene.

## Conclusion:

We show the feasibility of forensic assessment from skeletonized remains despite challenges from incomplete recovery and decomposition artifacts. Identity determination succeeded in most cases through anthropological methods, DNA and personal effects, though cause/manner of death remained elusive due to skeletal damage. Multidisciplinary collaboration, improved infrastructure and scene correlation are essential to addressing forensic gaps in resource-limited, developing-country settings.

## Statements and declarations:

Not Applicable
